# Time to Cancer Treatment and Chemotherapy Relative Dose Intensity for Patients With Breast Cancer Living With HIV

**DOI:** 10.1001/jamanetworkopen.2023.46223

**Published:** 2023-12-05

**Authors:** Daniel S. O’Neil, Yehoda M. Martei, Katherine D. Crew, Brenda S. Castillo, Philippos Costa, Tristan Lim, Alissa Michel, Elizabeth Rubin, Neha Goel, Judith Hurley, Gilberto Lopes, Michael H. Antoni

**Affiliations:** 1 Section of Medical Oncology, Department of Internal Medicine, Yale School of Medicine, New Haven, Connecticut; 2 Yale Cancer Center, Yale School of Medicine, New Haven, Connecticut; 3 Department of Medicine (Hematology-Oncology), University of Pennsylvania, Philadelphia; 4 Abramson Cancer Center, Perelman School of Medicine, Philadelphia, Pennsylvania; 5 Division of Hematology/Oncology, Department of Medicine, Vagelos College of Physicians and Surgeons, New York, New York; 6 Herbert Irving Comprehensive Cancer Center, Columbia University Irving Medical Center, New York, New York; 7 Department of Internal Medicine, Hospital of the University of Pennsylvania, Philadelphia; 8 Perelman School of Medicine, University of Pennsylvania, Philadelphia; 9 Memorial Cancer Institute, Memorial Healthcare System, Hollywood, Florida; 10 Division of Surgical Oncology, DeWitt Daughtry Family Department of Surgery, University of Miami Miller School of Medicine, Miami, Florida; 11 Sylvester Comprehensive Cancer Center, University of Miami Health System, Miami, Florida; 12 Division of Medical Oncology, Department of Medicine, University of Miami Miller School of Medicine, Miami, Florida; 13 Department of Psychiatry and Behavioral Sciences, University of Miami Miller School of Medicine, Miami, Florida

## Abstract

**Importance:**

Patients with breast cancer and comorbid HIV experience higher mortality than other patients with breast cancer.

**Objective:**

To compare time to cancer treatment initiation and relative dose intensity (RDI) of neoadjuvant and adjuvant chemotherapy among patients with breast cancer with vs without HIV.

**Design, Setting, and Participants:**

A retrospective, matched cohort study enrolled women who received a diagnosis of breast cancer from January 1, 2000, through December 31, 2018. The electronic medical records of 3 urban, academic cancer centers were searched for women with confirmed HIV infection prior to or simultaneous with diagnosis of stage I to III breast cancer. Tumor registry data were used to identify 2 control patients with breast cancer without HIV for each participant with HIV, matching for study site, stage, and year of cancer diagnosis. Statistical analysis was performed from December 2022 to October 2023.

**Exposure:**

HIV infection detected before or within 90 days of participants’ breast cancer diagnosis.

**Main Outcomes and Measures:**

The primary outcome was time to breast cancer treatment initiation, defined as the number of days between cancer diagnosis and first treatment. The secondary outcome was overall RDI for patients who received chemotherapy. These outcomes were compared by HIV status using Cox proportional hazards regression and linear regression modeling, respectively, adjusting for confounding demographic and clinical factors. Exploratory outcomes included instances of anemia, neutropenia, thrombocytopenia, and liver function test result abnormalities during chemotherapy, which were compared using Fisher exact tests.

**Results:**

The study enrolled 66 women with comorbid breast cancer and HIV (median age, 51.1 years [IQR, 45.7-58.2 years]) and 132 with breast cancer alone (median age, 53.9 years [IQR, 47.0-62.5 years]). The median time to first cancer treatment was not significantly higher among patients with HIV than those without (48.5 days [IQR, 32.0-67.0 days] vs 42.5 days [IQR, 25.0-59.0 days]; adjusted hazard ratio, 0.78, 95% CI, 0.55-1.12). Among the 36 women with HIV and 62 women without HIV who received chemotherapy, the median overall RDI was lower for those with HIV vs without HIV (0.87 [IQR, 0.74-0.97] vs 0.96 [IQR, 0.88-1.00]; adjusted *P* = .01). Grade 3 or higher neutropenia during chemotherapy occurred among more women with HIV than those without HIV (13 of 36 [36.1%] vs 5 of 58 [8.6%]).

**Conclusions and Relevance:**

This matched cohort study suggests that patients with breast cancer and HIV may have experienced reduced adjuvant chemotherapy RDI, reflecting greater dose reductions, delays, or discontinuation. Strategies for supporting this vulnerable population during chemotherapy treatment are necessary.

## Key Points

**Question:**

Do patients with breast cancer and comorbid HIV infection experience differences in their cancer care compared with patients without HIV infection?

**Findings:**

In this matched-cohort study including 198 women from 3 academic cancer centers, patients with breast cancer and HIV had no significant difference in time to initiate cancer treatment. Among patients who received neoadjuvant or adjuvant chemotherapy, those with comorbid HIV had a median chemotherapy relative dose intensity (RDI) of 0.87, and those without HIV had a median RDI of 0.96.

**Meaning:**

This study suggests that patients with breast cancer and comorbid HIV do not face greater delays in starting cancer treatment but are more likely to experience suboptimal delivery of adjuvant chemotherapy.

## Teaser

This matched cohort study compares time to cancer treatment initiation and relative dose intensity of neoadjuvant and adjuvant chemotherapy among patients with breast cancer with vs without HIV.

## Introduction

With antiretroviral therapy, life expectancy among people living with HIV in the US increased from 10.5 years in 1996 to 28.9 years in 2011.^1,2^ Accordingly, the population of people living with HIV is aging. In 2016, 58% of people living with HIV in the US were 45 years of age or older.^3^ Older people living with HIV are at risk for non-AIDS–defining cancers, and between 1991 to 1995 and 2001 to 2005, there was a 10-fold increase in breast cancer cases among people in the US living with AIDS.^4^

Women living with HIV (WLHIV) do not seem to be at an increased risk of developing breast cancer.^5,6^ However, once they have received a diagnosis of breast cancer, WLHIV have worse outcomes. Analyses of the National Cancer Database; Surveillance, Epidemiology, and End Results (SEER)–Medicare; and the US-based HIV/AIDS Cancer Match Study registries demonstrate significantly worse cancer-specific mortality (hazard ratios [HRs] from 1.85 to 2.64) and overall mortality (HR, 1.85) for patients with breast cancer living with HIV.^7,8,9^

The causes of these survival disparities are likely multifactorial, including the possibility that patients with breast cancer living with HIV are more likely to receive substandard cancer care. The prevalence rates of HIV are higher among racial and ethnic groups that have historically faced reduced access to high-quality cancer care.^3,10,11,12,13,14,15^ Alternatively, longstanding HIV infection and its treatment may be associated with reduced tolerance for effective but toxic cancer therapies among WLHIV.^16,17,18^

Attempts to study breast cancer care quality among WLHIV have been limited by the relatively few patients with both diagnoses treated at any individual cancer center or the inherent limitations of larger data sets, which lack granular treatment data. Analysis from the SEER-Medicare registry shows that patients with cancer living with HIV experienced a longer delay between cancer diagnosis and first treatment (42.5 vs 36 days) but that analysis included only women aged 65 years or older and just 12 patients with breast cancer living with HIV.^19^ Conflicting reports from Botswana and South Africa suggest that WLHIV may or may not be less tolerant of chemotherapies used for breast cancer, but even patients without HIV in those studies received a lower chemotherapy dose intensity than is typically given in the US.^20,21^

We aimed to overcome these challenges to studying the associations between comorbid HIV and breast cancer care by pooling retrospective data from 3 large, urban cancer centers. Specifically, we assessed associations between HIV infection and 2 care quality indicators: time to treatment initiation after cancer diagnosis and neoadjuvant and adjuvant chemotherapy relative dose intensity (RDI).

## Methods

The institutional review boards of each participating university (Sylvester Comprehensive Cancer Center of the University of Miami Miller Health System, Abramson Cancer Center of Penn Medicine, and Herbert Irving Comprehensive Cancer Center of Columbia University) approved this study and issued waivers of consent for the collection of retrospective, deidentified patient data. Shared data were fully deidentified and used within the scope of negotiated data sharing agreements. This report followed the Strengthening the Reporting of Observational Studies in Epidemiology (STROBE) reporting guideline.^22^

### Study Sites and Participants

Study participant data were drawn from 3 urban, academic cancer systems: the Sylvester Comprehensive Cancer Center of the University of Miami Miller Health System, including women treated at Jackson Memorial Hospital, in Miami, Florida; the Abramson Cancer Center of Penn Medicine in Philadelphia, Pennsylvania; and the Herbert Irving Comprehensive Cancer Center of Columbia University in New York City.

The electronic medical record systems at each site were queried for records containing at least 1 respective *International Classification of Diseases, Ninth Revision* or *International Statistical Classification of Diseases and Related Health Problems, Tenth Revision* code that pertained to each case of breast cancer (eTable 1 in Supplement 1) and to HIV (eTable 2 in Supplement 1). The list of potential participants identified through this process was manually reviewed by the investigators to identify individuals meeting the following eligibility criteria: (1) female sex; (2) aged 18 years or older; (3) histologically confirmed invasive breast cancer first diagnosed between January 1, 2000, and December 31, 2018; (4) breast cancer stage between I and III at presentation; and (5) confirmed HIV infection diagnosed no later than 90 days after the initial breast cancer diagnosis. HIV infection was deemed confirmed for inclusion in this study by the presence of a positive serology test result, a quantifiable HIV viral load, or a clinician note clearly referencing an HIV infection. Eligibility did not require patients to have received all their care at the study site, but those without available data on the timing of cancer diagnosis or treatment initiation were excluded, as were women whose first cancer therapy occurred as part of a clinical trial. Participants meeting the inclusion criteria were enrolled and comprised the WLHIV group.

Investigators at each site then used data from their institutional cancer registries and the MatchIt package in R, version 3.3.0 (R Project for Statistical Computing), to generate a list of 12 other patients with breast cancer matched to each member of the WLHIV group by disease stage and year of diagnosis.^23^ Participants from Miami, Florida, were also matched based on receipt of care within the university’s clinics vs the public hospital. Investigators manually reviewed the medical records of the matched patients for women meeting study inclusion and exclusion criteria other than comorbid HIV infection until 2 matching participants without HIV infection were identified for each participant in the WLHIV group. These additional participants comprised the control group. In both groups, we also identified a subcohort of participants who had received initial neoadjuvant or adjuvant chemotherapy for their breast cancer at the enrolling study site.

### Data Collection and Outcomes

The electronic medical records of participants from both groups were reviewed, and data were manually extracted to identical REDCap databases hosted at each institution.^24^ Data were collected on participants’ demographic characteristics (ie, age at time of breast cancer diagnosis, self-reported race and ethnicity, relationship status, and country of birth), breast cancer (ie, stage, hormone receptor status, *ERBB2* status, grade, and date of first breast cancer–directed surgery, endocrine therapy, chemotherapy, and radiotherapy), and, if available, HIV (ie, date of diagnosis and medical regimen at the time of breast cancer diagnosis). In the subset of participants who received chemotherapy at a study site, data were also collected on that first-line regimen (ie, specific drugs prescribed and dates and dose amounts of each drug actually received by the participants) and any available laboratory test data immediately before and during treatment (ie, hemoglobin concentration, absolute neutrophil count, platelet count, alanine and aspartate transaminase concentrations, alkaline phosphatase concentrations, and total bilirubin concentrations). Date of death was captured for participants known to be deceased. If a participant had no medical record entries later than December 31, 2019, date of last documented contact with the clinic was recorded.

Our primary outcome was time to breast cancer treatment initiation, which was defined as the number of days from the date of histologically confirmed breast cancer diagnosis and the receipt of the first breast cancer–directed therapy of any modality. This outcome was calculated for all participants in both groups.

Our secondary outcome was overall chemotherapy RDI, which was measured only in the subcohort of participants who received their initial chemotherapy at the 3 hospital systems participating in this study. The RDI of each individual prescribed drug was calculated separately using the following standard formula^25^: (Sum of all delivered doses/total number of days to deliver all doses given)/(total standard dose/total number of days to deliver full standard dose).

The standard dose amounts and the timing were based first on any information available in the prescribing clinicians’ notes. If no information was available from those notes, standards were based on available guidelines and literature describing the various regimens used. The overall RDI was the unweighted mean of the individual drug RDIs in the participant’s first regimen. Relative dose intensities within each drug class (ie, anthracyclines, cyclophosphamide, taxanes, fluorouracil, and platinums) were calculated in the same fashion. Doses that were completely missed due to early discontinuation or treatment abandonment were included in the numerator using a delivered dose of 0.0 mg and a standard delivery interval.

### Statistical Analysis

Statistical analysis was performed from December 2022 to October 2023. The characteristics for the full cohort of participants and the subcohort of participants in the chemotherapy RDI analysis were described using median (IQR) values and numbers and percentages. We compared the number of days to breast cancer treatment initiation between women with and women without comorbid HIV infection using a univariable Cox proportional hazards regression model. To control for demographic or clinical differences based on HIV status that might confound those results, we also constructed a multivariable Cox proportional hazards regression model of time to treatment initiation with covariates for HIV status, the matching factors of stage and year of diagnosis, and any other characteristic that was associated with the number days to treatment initiation, defined as a Wald test *P* ≤ .10 in individual univariable Cox proportional hazards regression models. With existing literature demonstrating that Black and Hispanic women face a higher risk than White women of receiving lower-quality breast cancer treatment,^26^ we used non-Hispanic White race as the common reference value for evaluating the association of self-reported race and ethnicity with the number of days to treatment initiation in our regression models.

In the chemotherapy subcohort, we compared the proportion in each group that received each drug class and the proportion in each group that received an overall RDI of 0.85 or higher (a dose intensity threshold shown to be associated with improved survival) using the Fisher exact test.^25,27,28^ We compared the median overall RDI and the median RDI of each drug class again using Wilcoxon rank sum testing. For the multivariable analysis of overall RDI, we constructed a linear regression model of participants’ continuous RDI values with covariates for HIV status, stage, year of diagnosis, and other characteristics associated with RDI in individual univariable logistic regression models (*P* ≤ .10).

To explore causes of differences in RDI, we described the proportion of participants in each arm experiencing Common Terminology Criteria for Adverse Events, version 5.0,^29^ grade 3 or higher neutropenia, grade 3 or higher anemia, grade 1 or higher thrombocytopenia, and grade 1 or higher hepatic toxic effects (transaminitis, alkaline phosphatase elevation, or hyperbilirubinemia) during chemotherapy and compared arms using Fisher exact tests.

As an exploratory analysis, we also compared overall survival from time of breast cancer diagnosis using Kaplan-Meier curves and log-rank testing.^30^ Participants not documented to be deceased were censored on the date of their lasted documented contact with the clinic or, if still in follow-up, administratively censored on December 31, 2019.

All tests were 2-sided. A simple Bonferroni correction was used to account for multiple comparisons, and a regression-adjusted *P* ≤ .025 were considered significant for analysis of the primary and secondary outcomes. All other analyses and comparisons are considered exploratory. SAS, version 9.4 (SAS Institute Inc) was used for statistical testing.

## Results

Across the 3 institutions, we identified 66 eligible women with comorbid breast cancer and HIV: 38 (57.6%) from the University of Miami, 17 (25.8%) from the University of Pennsylvania, and 11 (16.7%) from Columbia University. With 2 matched controls for each woman living with HIV, we enrolled 132 control patients with breast cancer but without HIV (Table 1). The WLHIV were slightly younger at breast cancer diagnosis than the matched controls (median age, 51.1 years [IQR, 45.7-58.2 years] vs 53.9 years [IQR, 47.0-62.5 years]). A higher proportion of the WLHIV were of non-Hispanic Black race (43 of 66 [65.2%] vs 26 of 132 [19.7%]) and reported being either single, divorced, or separated (48 of 66 [72.7%] vs 72 of 132 [54.6%]). A smaller proportion of WLHIV had hormone receptor–positive cancer (45 of 66 [68.2%] vs 114 of 132 [86.4%]). The chemotherapy subcohort included 36 WLHIV and 62 women without HIV; differences in race and relationship status persisted in that subcohort.

**Table 1.** Participant Characteristics for the Full Study Cohort and Chemotherapy Subcohort

Characteristic	Full cohort, No. (%)		Chemotherapy subcohort, No. (%)	
	Without HIV (n = 132)	With HIV (n = 66)	Without HIV (n = 62)	With HIV (n = 36)
Age, median (IQR), y	53.9 (47.0-62.5)	51.1 (45.7-58.2)	49.8 (43.6-58.0)	51.1 (45.19-58.2)
Race and ethnicity				
Asian or Pacific Islander	5 (3.8)	0	2 (3.2)	0
Hispanic	57 (43.2)	13 (19.7)	34 (54.8)	7 (19.4)
Non-Hispanic Black	26 (19.7)	43 (65.2)	16 (25.8)	24 (66.7)
Non-Hispanic White	42 (31.8)	9 (13.6)	10 (16.1)	5 (13.9)
Unknown	2 (1.5)	1 (1.5)	0	0
Relationship status				
Single, divorced, or separated	72 (54.6)	48 (72.7)	38 (61.3)	27 (75.0)
Married or partnered	58 (43.9)	9 (13.6)	24 (38.7)	2 (5.6)
Unknown	2 (1.5)	9 (13.6)	0	7 (19.4)
Country of origin				
US	58 (43.9)	27 (40.9)	21 (33.9)	13 (36.1)
Other	35 (26.5)	12 (18.2)	23 (37.1)	8 (22.2)
Unknown	39 (29.6)	27 (40.9)	18 (29.0)	15 (41.7)
Site				
Miami	76 (57.6)	38 (57.6)	43 (69.4)	22 (61.1)
Philadelphia	34 (25.8)	17 (25.8)	11 (17.7)	9 (25.0)
New York City	22 (16.7)	11 (16.7)	8 (12.9)	5 (13.9)
Stage				
I	44 (33.6)	25 (37.9)	7 (11.3)	4 (11.1)
II	58 (44.3)	30(45.5)	35 (56.5)	24 (66.7)
III	29 (22.1)	11 (16.7)	20 (32.3)	8 (22.2)
Hormone receptor status				
Positive	114 (86.4)	45 (68.2)	48 (77.4)	23 (63.9)
Negative	18 (13.6)	21 (31.8)	14 (22.6)	13 (36.1)
*ERBB2* status				
Positive	16 (12.1)	7 (10.6)	14 (22.6)	5 (13.9)
Negative	116 (87.9)	59 (89.4)	48 (77.4)	31 (86.1)
Grade				
1	18 (13.9)	9 (14.3)	3 (4.9)	3 (8.6)
2	53 (40.8)	18 (28.6)	27 (44.3)	12 (34.3)
3	45 (34.6)	23 (36.5)	25 (41.0)	15 (42.9)
Unknown	14 (10.8)	13 (20.6)	6 (9.8)	5 (14.3)
Year of breast cancer diagnosis				
2001-2005	12 (9.1)	10 (15.2)	8 (12.9)	5 (13.9)
2006-2009	26 (19.7)	10 (15.2)	9 (14.5)	6 (16.7)
2010-2014	50 (37.9)	24 (36.4)	20 (32.3)	13 (36.1)
2015-2018	44 (33.3)	22 (33.3)	25 (40.3)	12 (33.3)
First treatment modality				
Surgery	96 (75.6)	49 (75.4)	32 (53.3)	24 (66.7)
Chemotherapy	29 (22.8)	12 (18.5)	27 (45.0)	10 (27.8)
Endocrine therapy	2 (1.6)	3 (4.6)	1 (1.7)	2 (5.6)
Radiotherapy	0	1 (1.5)	0	0

In the full cohort, patients with breast cancer and comorbid HIV infection waited a median of 48.5 days (IQR, 32.0-67.0 days) from diagnosis for their first breast cancer treatment, while patients without HIV waited a median of 42.5 days (IQR, 25.0-59.0 days) (unadjusted HR, 0.73 [95% CI, 0.54-0.99]) (Table 2). When adjusting for differences in race and ethnicity, stage, grade, receipt of primary surgery, and year of cancer diagnosis between patients with and patients without HIV, HIV status was not significantly associated with a longer time to breast cancer treatment initiation (HR, 0.78 [95% CI, 0.55-1.12]). Non-Hispanic Black race and Hispanic ethnicity both remained associated with treatment initiation delays (HR, 0.50 [95% CI, 0.33-0.77] and HR, 0.67 [95% CI, 0.45-0.99], respectively).

**Table 2.** Factors Associated With Time to Breast Cancer Treatment Initiation

Covariate	Univariable HR (95% CI)	Unadjusted *P* value	Multivariable HR (95% CI)	Adjusted *P* value^a^
HIV infection (reference, uninfected)	0.73 (0.54-0.99)	.04	0.78 (0.55-1.12)	.18
Race and ethnicity (reference, non-Hispanic White)				
Asian or Pacific Islander	0.78 (0.31-1.95)	.59	0.56 (0.19-1.63)	.29
Hispanic	0.64 (0.45-0.92)	.02	0.67 (0.45-0.99)	.04
Non-Hispanic Black	0.45 (0.31-0.65)	<.001	0.50 (0.33-0.77)	.001
Stage (reference, stage I)				
Stage II	0.70 (0.51-0.97)	.03	0.81 (0.57-1.15)	.24
Stage III	0.64 (0.43-0.95)	.03	0.76 (0.47-1.23)	.26
Grade (reference, grade 3)				
Grade 1	1.79 (1.34-2.82)	.01	1.47 (0.91-2.35)	.11
Grade 2	1.20 (0.86-1.68)	.29	0.95 (0.67-1.36)	.79
Primary surgery	1.41 (1.01-1.97)	.046	1.35 (0.90-2.02)	.14
Year of diagnosis (reference, 2015-2018)				
2001-2005	0.99 (0.61-1.62)	.97	1.02 (0.60-1.74)	.95
2006-2009	1.01 (0.67-1.52)	.97	0.81 (0.52-1.26)	.35
2010-2014	1.11 (0.79-1.55)	.55	0.94 (0.66-1.36)	.76

Abbreviation: HR, hazard ratio.

^a^ From a Cox proportional hazards regression model including all listed covariates.

In the subcohort of patients receiving chemotherapy, fewer WLHIV were treated with a taxane (30 of 36 [83.3%] vs 61 of 62 [98.4%]) (Table 3). The median overall chemotherapy RDI was lower among WLHIV than patients without HIV (0.87 [IQR, 0.74-0.97] vs 0.96 [IQR, 0.88-1.00]; unadjusted *P* = .01), as was the proportion of patients receiving an overall RDI of 0.85 or higher (21 of 36 [58.3%] vs 51 of 62 [82.3%]; *P* = .02). Comparing the RDIs of individual drug types, WLHIV had lower recorded RDIs for anthracycline and taxane chemotherapies. In our multivariable comparison, White race met criteria for inclusion as a covariate along with HIV status and the original matching criteria (ie, stage and year of diagnosis). HIV status remained associated with lower overall RDI (β, −0.09 [95% CI, −0.15 to −0.03]; *P* = .01), while stage and year of diagnosis were not associated with lower overall RDI (Table 4). Unexpectedly, after adjustment for HIV status and other covariates, Black race showed a slight association with higher overall RDI (β, 0.09 [95% CI, 0.004-0.18]; *P* = .04). Testing the assumptions of linear regression for this model yielded concern for autocorrelation; adding a first-order autoregressive error correction parameter to our model resolved the evidence of autocorrelation without changing the significance or magnitude of HIV’s association with RDI. More myelotoxic effects were recorded among WLHIV as well, with increases in grade 3 or higher neutropenia (13 of 36 [36.1%] vs 5 of 58 [8.6%]; *P* = .002) (Table 5).

**Table 3.** Comparison of Chemotherapy Receipt by HIV Status

Characteristic	Patients without HIV (n = 62)	Patients with HIV (n = 36)	*P* value
Receipt of drug type, No. (%)			
Anthracyclines	39 (62.9)	25 (69.4)	.66^a^
Cyclophosphamide	54 (87.1)	29 (80.6)	.40^a^
Taxanes	61 (98.4)	30 (83.3)	.01^a^
Other (fluorouracil and platinums)	18 (29.0)	11 (30.6)	≥.99^a^
RDI			
Overall regimen, median (IQR)	0.96 (0.88-1.00)	0.87 (0.74-0.97)	.01^b^
Overall regimen RDI ≥0.85, No. (%)	51 (82.3)	21 (58.3)	.02^a^
Anthracyclines, median (IQR)	1.00 (0.95-1.00)	0.98 (0.83-1.00)	.02^b^
Cyclophosphamide, median (IQR)	1.00 (0.92-1.00)	0.98 (0.88-1.00)	.31^b^
Taxanes, median (IQR)	0.95 (0.86-1.00)	0.83 (0.62-0.95)	.006^b^
Other (fluorouracil and platinums), median (IQR)	0.97 (0.87-1.00)	0.83 (0.66-0.98)	.06^b^

Abbreviation: RDI, relative dose intensity.

^a^ Fisher exact test.

^b^ Wilcoxon rank sum test.

**Table 4.** Factors Associated With Neoadjuvant and Adjuvant Chemotherapy Relative Dose Intensity

Covariate	Univariable β coefficient (95% CI)	Univariable *P* value	Multivariable β coefficient (95% CI)	Adjusted *P* value^a^
HIV infection	−0.08 (−0.13 to −0.02)	.01	−0.09 (−0.15 to −0.03)	.01
Race and ethnicity (reference, non-Hispanic White)				
Asian or Pacific Islander	−0.004 (−0.21 to 0.20)	.97	0.03 (−0.18 to 0.24)	.75
Hispanic	0.03 (−0.02 to 0.09)	.25	0.07 (−0.02 to 0.15)	.12
Non-Hispanic Black	0.01 (−0.05 to 0.07)	.77	0.09 (0.004 to 0.18)	.04
Stage	−0.004 (−0.05 to 0.04)	.87	−0.005 (−0.05 to 0.04)	.84
Year of diagnosis	−0.01 (−0.04 to 0.02)	.48	−0.01 (−0.03 to 0.02)	.66

^a^ From a linear regression model including all listed covariates.

**Table 5.** Comparison of Chemotherapy-Related Toxic Effects

Toxic effect	No./total No. (%) of patients		*P* value^a^
	Without HIV	With HIV	
Neutropenia (grade ≥3)	5/58 (8.6)	13/36 (36.1)	.002
Anemia (grade ≥3)	7/59 (11.9)	8/37 (21.6)	.25
Thrombocytopenia (grade ≥1)	2/59 (3.4)	5/37 (13.5)	.10
Transaminitis (grade ≥1)	1/58 (1.7)	5/32 (15.6)	.02
Alkaline phosphatase elevation (grade ≥1)	0	1/32 (3.1)	.36
Hyperbilirubinemia (grade ≥1)	0	2/32 (6.3)	.12

^a^ Fisher exact test.

With a median follow-up of 69.4 months (IQR, 38.2-104.1 months) and a maximum follow-up of 190.6 months, the 5-year overall survival was 81.8% for WLHIV and 90.9% for patients without HIV (eFigure in Supplement 1). This was not a significant difference in this small group (HR, 1.73 [95% CI, 0.78-3.80]; log-rank *P* = .17).

## Discussion

In this multi-institutional cohort study of 66 patients with stage I to III breast cancer living with HIV and 132 matched patients with breast cancer without HIV, we did not find any significant differences in the time from breast cancer diagnosis to the start of cancer treatment after adjusting for non-Hispanic Black race and other covariates that differed based on HIV status. However, in the subcohort of patients who received chemotherapy, we did find that overall chemotherapy RDI was lower in the group of patients with HIV infection and that less than 60% of those women received an RDI of 0.85 or higher, a clinically relevant threshold that has shown prior association with survival.^25,27,28^ At the level of individual drugs, this association was especially pronounced for taxane agents. Decreased chemotherapy dose intensity was accompanied by an increased incidence of neutropenia. Differences in overall survival did not reach statistical significance in this small study, but the 73% increase in mortality among patients with breast cancer living with HIV is broadly consistent with larger registry-based and cohort studies from the US and sub-Saharan Africa.^7,8,9,31,32^

We hypothesized that WLHIV might experience large delays in receipt of cancer care stemming from either structural barriers to access or the need for more intensive pretreatment evaluation. Such delays were appreciable among patients in the SEER-Medicare database and, if present here, might contribute to poorer survival.^19,33,34^ However, WLHIV only waited 5.5 additional days to start cancer treatment, and this difference disappeared after adjusting for the greater proportion of Black and Hispanic women in the group living with HIV, suggesting that any difference may be driven by well-documented racial disparities in breast cancer care access.^35,36,37,38,39^ It is also unlikely that the modest difference measured here contributes substantially to survival disparities.

We also hypothesized that chemotherapy dosing would be delayed or reduced more often for WLHIV because of either reduced tolerance to chemotherapy-related toxic effects or less consistent adherence to the prescribed treatment schedule. Existing literature on chemotherapy dose intensity in patients with breast cancer living with HIV comes from sub-Saharan Africa. Botswanan patients with breast cancer did demonstrate a reduction in chemotherapy RDI associated with comorbid HIV infection, but the mean RDI for all patients was just 0.78.^20^ Conflicting results come from South Africa, where a large cohort study of breast cancer showed parity in RDI by HIV status and a median of 0.88 for all women.^21^ In the US academic setting, we found patients with breast cancer living with HIV reaching a median RDI comparable to South African WLHIV. However, in the US, this same dose intensity was associated with a disparity when compared with the nearly optimal level measured among patients with breast cancer without HIV. Myelosuppression is the most common cause of reduced RDI, and the increases in neutropenia among WLHIV, despite access to granulocyte colony stimulating factors, may explain some of the reduction seen here.^40^ Myelosuppression with chemotherapy may not only affect survival by reducing RDI; decreases in circulating CD4 cell count after cancer therapy have been independently associated with increased mortality among patients with cancer living with HIV.^41^

Differences in RDI were greatest during treatment with taxane chemotherapies. It is difficult to say whether these differences are due to an intrinsic intolerance to taxanes among WLHIV or the fact that taxanes are typically given during the second half of more aggressive protocols, when overall myelosuppressive effects have begun to accumulate. Overlapping metabolic pathways suggest a theoretical risk of drug-drug interactions with several common antiretroviral agents (eg, efavirenz, elvitegravir, and boosted protease inhibitors), but the same is true of cyclophosphamide, for which RDI did not differ.^42^ Peripheral neuropathy is an overlapping complication of chronic HIV infection. However, we cannot comment on whether WLHIV experienced more dose-limiting neuropathy than patients without HIV.

### Limitations

Our study has some limitations, including the small size of the cohort, instances of missing data, and the possibility of some data errors arising from manual data extraction. These limitations arose from the retrospective nature of this study and the need to pool data from diverse medical record systems to study a population that remains rare in the US. However, the association between HIV infection and chemotherapy tolerance reached statistical significance even in this small population, and our findings are both plausible and consistent with larger cohort studies from sub-Saharan Africa. Participants living with HIV often received their infectious disease care at other institutions, and data on CD4 counts, viral loads, prior opportunistic infections, and current therapies were frequently missing, which prevented analysis for interactions between HIV control and breast cancer care. All participants were receiving cancer care at large academic cancer centers, which could limit generalizability, but cancer care disparities seen at centers with experience treating patients with complex cancer are likely only exaggerated in community practices.

## Conclusions

To our knowledge, this cohort study consists of the largest collection of detailed cancer treatment data from US-based patients with breast cancer and comorbid HIV and is the first to show differences in chemotherapy tolerance among patients with breast cancer with or without HIV who were treated in a high-resource setting. Access to full medical record data provided more detail regarding cancer treatment timing and toxic effects than is available in tumor registries. Dose-limiting toxic effects and suboptimal receipt of neoadjuvant and adjuvant chemotherapy among patients with breast cancer living with comorbid HIV infection may contribute to this group’s well-documented increased risk of death compared with other women with breast cancer. Coinfection with HIV may also limit the ability to safely deliver other toxic but efficacious breast cancer treatments. Strategies to better support patients with breast cancer living with HIV during toxic therapies are needed to improve outcomes among this growing population.

## Notes

Supplement 1. **eTable 1.** International Statistical Classification of Diseases and Related Health Problems (ICD)-9 and ICD-10 Billing Codes Used to Identify Potential Participants With Breast Cancer **eTable 2.** International Statistical Classification of Diseases and Related Health Problems (ICD)-9 and ICD-10 Billing Codes Used to Identify Potential Participants With HIV **eFigure.** Overall Survival Stratified by HIV Infection Status

Supplement 2.
Data Sharing Statement
