# Supplementary material for: Time to Cancer Treatment and Chemotherapy Relative Dose Intensity for Patients With Breast Cancer Living With HIV
Source: JAMA Netw Open. 2023 Dec 5;6(12):e2346223. doi: 10.1001/jamanetworkopen.2023.46223 (PMC10698616; doi:10.1001/jamanetworkopen.2023.46223)
Supplement: Supplement 1. — eTable 1. International Statistical Classification of Diseases and Related Health Problems (ICD)-9 and ICD-10 Billing Codes Used to Identify Potential Participants With Breast Cancer eTable 2. International Statistical Classification of Diseases and Related Health Problems (ICD)-9 and ICD-10 Billing Codes Used to Identify Potential Participants With HIV eFigure. Overall Survival Stratified by HIV Infection Status [file jamanetwopen-e2346223-s001.pdf]

Supplementary Online Content

O'Neil DS, Martei YM, Crew KD, et al. Time to cancer treatment and chemotherapy relative dose intensity for patients with breast cancer living with HIV. *JAMA Netw Open.* 2023;6(12):e2346223. doi:10.1001/jamanetworkopen.2023.46223

eTable 1. International Statistical Classification of Diseases and Related Health Problems (ICD)-9 and ICD-10 Billing Codes Used to Identify Potential Participants With Breast Cancer

eTable 2. International Statistical Classification of Diseases and Related Health Problems (ICD)-9 and ICD-10 Billing Codes Used to Identify Potential Participants With HIV

eFigure. Overall Survival Stratified by HIV Infection Status

This supplementary material has been provided by the authors to give readers additional information about their work.

eTable 1. International Statistical Classification of Diseases and Related Health Problems (ICD)-9 and ICD-10 Billing Codes Used to Identify Potential Participants With Breast Cancer

ICD-9 Codes	ICD-10 Codes
• 174.X<br>• V10.3<br>• 233.0	• C50.XX<br>• Z17.X<br>• Z85.3<br>• D05.1X

© 2023 O'Neil DS et al. *JAMA Network Open.*

eTable 2. International Statistical Classification of Diseases and Related Health Problems (ICD)-9 and ICD-10 Billing Codes Used to Identify Potential Participants With HIV

ICD-9	ICD-10
• 042<br>• V08<br>• 079.53<br>• 795.71<br>• 647.6X	• B20.X<br>• B21.X<br>• B22.X<br>• B23.X<br>• B24<br>• B97.35<br>• R75<br>• Z21

© 2023 O'Neil DS et al. *JAMA Network Open.*

eFigure. Overall Survival Stratified by HIV Infection Status

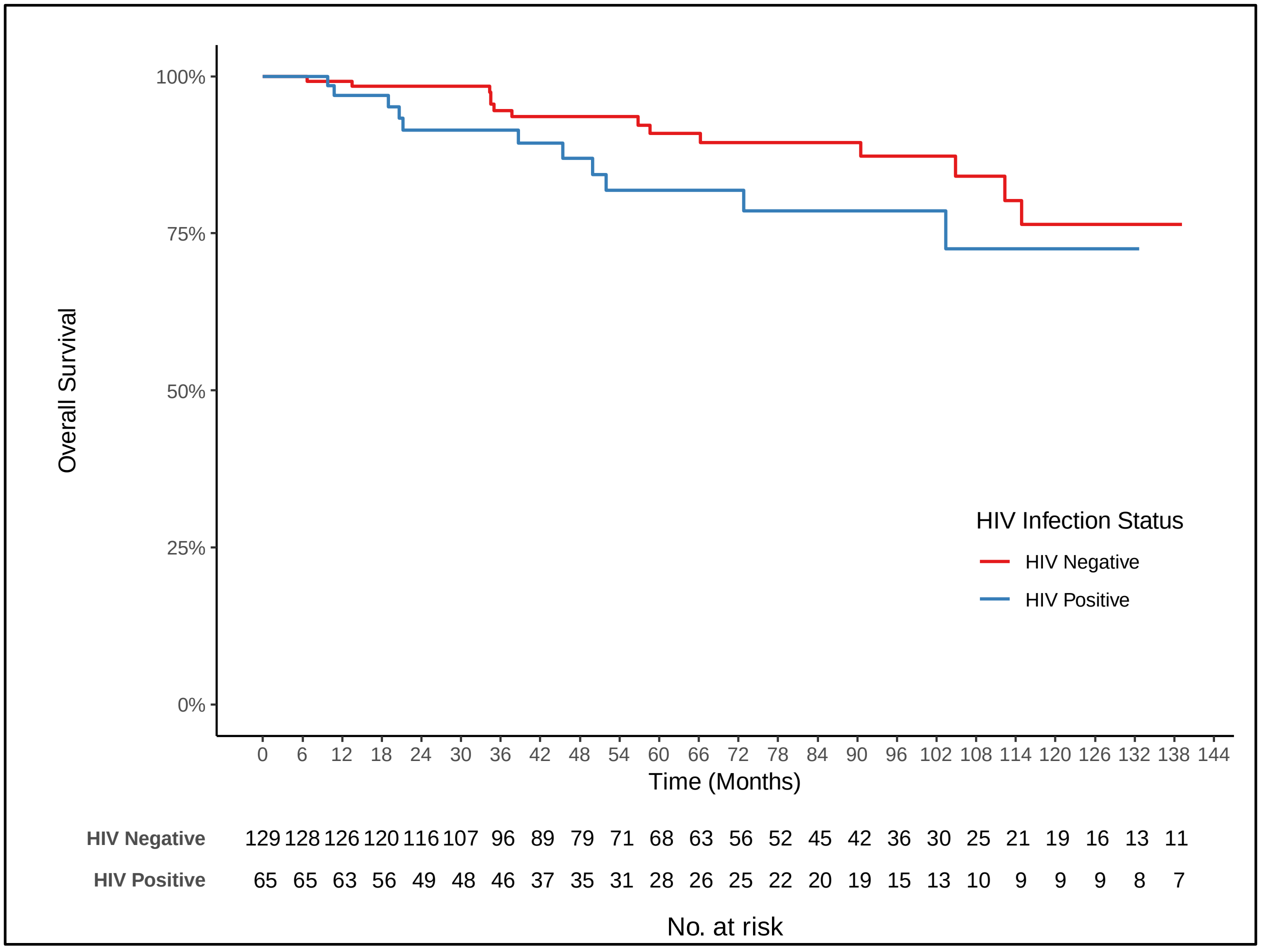

© 2023 O'Neil DS et al. *JAMA Network Open.*
