# Supplementary material for: Time to Cancer Treatment and Chemotherapy Relative Dose Intensity for Patients With Breast Cancer Living With HIV
Source: JAMA Netw Open. 2023 Dec 5;6(12):e2346223. doi: 10.1001/jamanetworkopen.2023.46223 (PMC10698616; doi:10.1001/jamanetworkopen.2023.46223)
Supplement: Supplement 2. — Data Sharing Statement [file jamanetwopen-e2346223-s002.pdf]

Data Sharing Statement

O’Neil. Time to Cancer Treatment and Chemotherapy Relative Dose Intensity for Patients With Breast Cancer Living With HIV. *JAMA Netw Open*. Published December 05, 2023. doi:10.1001/jamanetworkopen.2023.46223

Data
Data available: Yes
Data types: Other (please specify)
Additional Information: The data underlying this article were provided by the participating medical centers under respective data use agreements. Data will be shared on request to the corresponding author to the extent that is permissible according to those agreements.
How to access data: daniel.oneil@yale.edu
When available: With publication

Supporting Documents
Document types: None

Additional Information
Who can access the data: Data will be shared on request to the corresponding author to the extent that is permissible according to those agreements.
Types of analyses: For any purpose
Mechanisms of data availability: With a signed data access agreement
